# Full-time hospitalization in child and adolescent psychiatry: an overlook of the Tunisian situation

**DOI:** 10.1192/j.eurpsy.2023.479

**Published:** 2023-07-19

**Authors:** L. Sahli, Z. Abbes, S. Halayem, A. Bouden

**Affiliations:** child and adolescent Psychiatry, Razi Hospital, Manouba, Tunisia

## Abstract

**Introduction:**

Admissions in a child psychiatry unit can be voluntary or unvoluntary in some cases when the patient meets specific criteria.

**Objectives:**

The aim of our study was to assess the frequency and trend over time of admissions of minors in the child psychiatry department in Razi Hospital between 2011 and 2019, and to examine the psychiatric diagnoses in involuntary admissions of minors.

**Methods:**

We conducted a retrospective study of medical records of inpatients admitted to the hospitalization unit of the child psychiatry department in Razi Hospital in Tunisia between 2011 and 2019.

**Results:**

Over the nine years, the total number of hospitalizations was 924. There is a slight female predominance over the total number of hospitalizations (sex ratio = 0.85). There was no consistent and significant change in the number of hospitalizations between 2011 and 2019. A growing increase in the number of compulsory hospitalizations was noted. From 2011 to 2019, the number of compulsory admissions increased from 03 in 2011 to 22 in 2019.

Regarding compulsory hospitalizations, admission requests came from child protection delegates, public prosecutors or family judges. Conduct disorder was found in 33.3% of the cases followed by a normal psychiatric examination in 11.8% of the cases. Mood disorders were found in 9.8% of the cases.

**Conclusions:**

Our study shows the explosion in the number of compulsory hospitalizations despite a relatively stable total number of hospitalizations. More comprehensive guidance for legal authorities is needed regarding the compulsory admission of minors.

**Disclosure of Interest:**

None Declared

